# Detection of Hepatitis E Antibodies in Kazakhstan: A Pilot Study

**DOI:** 10.5195/cajgh.2018.324

**Published:** 2018-02-22

**Authors:** Francesca Cainelli, Gonzalo Hortelano, Baurzhan Negmetzhanov, Aigerim Ibrayeva, Kulpash Kaliaskarova, Denis Bulanin, Sandro Vento

**Affiliations:** 1 Department of Medicine, School of Medicine, Nazarbayev University, Astana, Kazakhstan; 2 Department of Biology, School of Science and Technology, Nazarbayev University, Astana, Kazakhstan; 3 Department of Biomedical Sciences, School of Medicine, Nazarbayev University, Astana, Kazakhstan; 4 Hepatology Coordinating Center, Ministry of Health, Astana, Kazakhstan; 5 University Medical Center, Astana, Kazakhstan

**Keywords:** Hepatitis E, anti-HEV antibodies, Kazakhstan

## Abstract

**Introduction:**

Hepatitis E virus exposure is associated with sporadic cases of acute hepatitis and outbreaks in many countries worldwide. It is particularly dangerous for pregnant women, in whom the mortality rate is high. There are no previously published data reporting circulation of this virus in Kazakhstan.

**Methods:**

We tested blood samples for IgG anti-hepatitis E virus antibodies in 199 Kazakh participants; of these 119 were workers at the EXPO 2017 building site in Astana, 35 were volunteers who got tested at the Astana City Hall on the World Hepatitis Day 2017, and 45 were volunteers who presented for screening at the Hepatogastroenterology Outpatient Clinic of the Republican Diagnostic Center, University Medical Center.

**Results:**

11 (5.5%) individuals were positive for IgG anti-HEV antibodies, with a higher seroprevalence in males (7; 6.8%) vs females (4; 4.5%). The highest number of positive samples was in the 32–46 years age group.

**Conclusions:**

This pilot study suggests that Hepatitis E virus has been circulating in Kazakhstan. Studies are needed to determine whether it continues to be present, which viral genotypes are involved and what are the best methodologies for preventing its spread.

Hepatitis E virus (HEV) is the most recently discovered major hepatitis virus, which poses a significant threat to pregnant women. Its existence was first suggested in 1980 by Khuroo, who studied a non-A, non-B hepatitis outbreak in the Kashmir Valley (India),^1^ and its genome was entirely cloned in 1991.^2^ HEV infection causes acute sporadic hepatitis and outbreaks worldwide. The virus has four genotypes. HEV 1 and 2 infect humans, are found largely in low and middle-income countries, and are transmitted through the fecal-oral route (mainly through contaminated water). Genotypes 3 and 4 are found mainly in high-income countries, infect humans as well as animals, and cause sporadic cases of acute hepatitis. HEV constitutes an important health problem in low- and middle-income countries, as acute sporadic form of hepatitis E is nowadays likely the most frequent cause of acute viral hepatitis worldwide.^3^ The worldwide incidence of acute genotypes 1 and 2 HEV infection was estimated in 2005 to be around 20.1 million cases, with 3.4 million symptomatic cases, 70,000 deaths, and 3,000 stillbirths.^4^

Although HEV infection usually causes self-limiting acute hepatitis with a low mortality rate, fulminant hepatitis frequently develops in pregnant women, especially at late gestational stages, and leads to a high maternal mortality rate (up to 31%) in developing countries.^5^,^6^,^7^ HEV-infected pregnant women develop obstetric complications, including intrauterine fetal death, preterm delivery, and stillbirth much more frequently than non-HEV-infected pregnant women.^8^ Interestingly, during community outbreaks of acute hepatitis E, clinical attack rates were higher among adolescents and young adults than among children or older adults.^9^ In patients with chronic liver disease of other etiology, acute HEV infection can determine flare ups of liver disease, and cause decompensation or liver failure in previously compensated cirrhotic patients.^10^

HEV can also cause chronic hepatitis in immunosuppressed patients, including transplant recipients, human immunodeficiency virus-infected individuals with low CD4 cell count, and patients with hematological malignancies treated with chemotherapy.^10^

Neurologic complications have been observed in large number of patients with HEV infection,^11^ as well as renal and rheumatologic manifestations in a smaller number of cases.

Outbreaks of HEV infection can occur in community-based settings (villages, cities, or provinces), military units, colleges, prisons, factories, and cruise ships. Outbreaks have been reported in Central Asian countries, specifically in Kyrgyzstan,^12^ Uzbekistan,^13^ and Turkmenistan;^14^ the latter was particularly large, occurred in the Dashoguz province and affected over 16,000 people.^14^ In Kazakhstan, a likely outbreak of acute hepatitis E occurred during the 1950s in the South, where case fatality rate reached 10% in hospitalized pregnant women.^15^ Currently, tests for IgM anti-HEV antibodies are performed in pregnant women in some Infectious Diseases Hospitals in Kazakhstan, but no results have been published to date.

We have performed a cross sectional study and tested for anti-HEV antibodies (HEV IgG 3rd generation EIA, DIA.PRO, Sesto San Giovanni, Italy) 199 Kazakh participants who gave their informed consent. Participants included 119 workers at the EXPO 2017 building site in Astana who presented consecutively in one day, 35 volunteers at the Astana City Hall on the World Hepatitis Day 2017, and 45 volunteers who presented consecutively for screening over a period of ten months at the Hepatogastroenterology Outpatient Clinic of the Republican Diagnostic Center, University Medical Center (UMC). The participants were identified to be Kazakh residents in the country either by their employer (in the case of the EXPO 2017 site) or by the personnel working at the City Hall who contributed to the event promotion or by the healthcare staff working at the Hepatogastroenterology Outpatient Clinic of the Republican Diagnostic Center, University Medical Center (UMC). Selection of these three sites were based on the idea of screening diverse representation of different population groups of Kazakhstan. All participants were briefed about the voluntary nature of the study and its objectives. The Institutional Review Boards of both Nazarbayev University and UMC granted approval for this study.

5 mL of blood were collected from each individual; serum was separated and stored at −80°C until used in the ELISA test. Specimens were considered positive at a cut off value ≥ 1, as per manufacturer’s instructions.

We collected information about age, gender, place (city, town, or village) of residence, history of jaundice, any previous diagnosis of hepatitis (if yes type was recorded as A, B, C, D or E), and alcohol consumption (current and past) using paper surveys. 103 participants were men and 89 women; in seven cases the gender was unreported (i.e. not indicated in the data sheets filled by the tested individuals). Age was available for 194 individuals (mean 39.3 years, range 9 – 77).

Overall, 11 (5.5%) individuals sampled were positive for anti-HEV antibodies; with higher prevalence among males (7 individuals; 6.8%) compared to females (4 individuals; 4.5%). No positivity was observed in the oldest age groups (over 60 years of age), while the highest number of positive samples were identified in the group between 32 and 46 years of age.

In the future, we plan to test all patients with acute and fulminant hepatitis admitted to hospitals in Astana to verify whether HEV is the etiological factors of some of these cases. Should this be the case, we will establish the genotypes of HEV that are found in Kazakhstan. The identification of genotypes will allow characterizing whether only genotypes HEV-1 and HEV-2 or also genotypes HEV-3 and HEV-4 circulate in Kazakhstan. Preventive measures can then eventually be instituted to limit the possible circulation of the hepatitis E virus in the country and nearby regions.
